# Enamel Anti-Demineralization Effect of Orthodontic Adhesive Containing Bioactive Glass and Graphene Oxide: An In-Vitro Study

**DOI:** 10.3390/ma11091728

**Published:** 2018-09-14

**Authors:** Seung-Min Lee, Kyung-Hyeon Yoo, Seog-Young Yoon, In-Ryoung Kim, Bong-Soo Park, Woo-Sung Son, Ching-Chang Ko, Sung-Ae Son, Yong-Il Kim

**Affiliations:** 1 Department of Orthodontics, Dental Research Institute, Pusan National University Dental Hospital, Yangsan 50612, Korea; seungminlee@pusan.ac.kr (S.-M.L.); wsson@pusan.ac.kr (W.-S.S.); 2 School of Materials Science and Engineering, Pusan National University, Busan 46241, Korea; seweet07@pusan.ac.kr (K.-H.Y.); syy3@pusan.ac.kr (S.-Y.Y.); 3 Department of Oral Anatomy, School of Dentistry, Pusan National University, Yangsan 50612, Korea; biowool@pusan.ac.kr (I.-R.K.); parkbs@pusan.ac.kr (B.-S.P.); 4 Department of Orthodontics, School of Dentistry, University of North Carolina at Chapel Hill, Chapel Hill, NC 27516, USA; Ching-Chang_Ko@unc.edu; 5 Department of Conservative Dentistry, School of Dentistry, Pusan National University, Yangsan 50612, Korea; 6 Institute of Translational Dental Sciences, Pusan National University, Busan 46241, Korea

**Keywords:** anti-demineralization, antibacterial effect, white spot lesion, graphene oxide, bioactive glass

## Abstract

White spot lesions (WSLs), a side effect of orthodontic treatment, can result in reversible and unaesthetic results. Graphene oxide (GO) with a bioactive glass (BAG) mixture (BAG@GO) was added to Low-Viscosity Transbond XT (LV) in a ratio of 1, 3, and 5%. The composite’s characterization and its physical and biological properties were verified with scanning electron microscopy (SEM) and X-ray diffraction (XRD); its microhardness, shear bond strength (SBS), cell viability, and adhesive remnant index (ARI) were also assessed. Efficiency in reducing WSL was evaluated using antibacterial activity of *S. mutans*. Anti-demineralization was analyzed using a cycle of the acid-base solution. Adhesives with 3 wt.% or 5 wt.% of BAG@GO showed significant increase in microhardness compared with LV. The sample and LV groups showed no significant differences in SBS or ARI. The cell viability test confirmed that none of the sample groups showed higher toxicity compared to the LV group. Antibacterial activity was higher in the 48-h group than in the 24 h group; the 48 h test showed that BAG@GO had a high antibacterial effect, which was more pronounced in 5 wt.% of BAG@GO. Anti-demineralization effect was higher in the BAG@GO-group than in the LV-group; the higher the BAG@GO concentration, the higher the anti-demineralization effect.

## 1. Introduction

Orthodontic treatment is performed to improve both functionality and aesthetics, but undesirable side effects can often occur. White spot lesions (WSLs) are one of the side effects of orthodontic treatment. The white and chalky surface of WSLs is a result of demineralization of the enamel surface. Fejerskov and Kidd defined WSLs as the “first sign of a caries lesion on enamel that can be detected with the naked eye” [1]. The opaque surface of WSLs can be easily detected with the naked eye, and WSLs on anterior teeth is undesirable, because one of the goals of orthodontic treatment is the aesthetic outcome. The main cause of WSLs, or demineralization, is a decrease in intraoral pH due to bacterial activity. Orthodontic fixed appliances create an irregular surface on teeth, which can then serve as an accumulation site for plaque [2]. *Streptococcus mutans* and *Lactobacilli*, the main pathogens of dental caries, decrease intraoral pH [3]. This effect, combined with an irregular enamel surface area and poor oral hygiene during orthodontic treatment, precipitates enamel demineralization. Patient education and regular fluoride application have been utilized to prevent WSLs. In recent years, researchers have been carrying out supplementation of bonding agents with biomaterials to prevent WSLs. Biomaterials added to bonding agents should be harmless to the human body, shouldn’t interfere with the physical properties of bonding agent, and should hinder the growth of bacteria that cause demineralization by either decreasing the pH or releasing ions to increase the pH. One of the biomaterials being studied contemporarily is Bioactive glass (BAG), with the basic structure of Si-O-Si, and consisting of CaO, Na_2_O, and P_2_O_2_. BAG functions as a filler and exhibits both an antibacterial function and a buffer effect (by releasing ions when added to resin pastes) [4]. In a liquid environment, BAG releases Na^+^, Ca2^+^, and PO_4_^3−^ ions and develops into a super-saturated ion state. On enamel surfaces, released ions convert precipitated amorphous calcium phosphate layer into apatite. BAG exerts its antibacterial effect by increasing intraoral pH through ion-release, and therefore, compensating for the decrease in pH caused by oral bacteria [5,6]. Graphene and graphene-based materials are recognized as biomaterials in dental fields such as tissue engineering. Graphene oxide (GO) is a carbon-based plate structure, and it has been studied for an extended period in the tissue engineering field, as an osteoinductive factor [7,8]. The antibacterial effect of GO, especially on dental pathogens, has been discovered recently, and it has been shown that GO displays the highest antibacterial effect in comparison to other graphene-based materials such as graphite, graphite oxide, graphene oxide, and reduced graphene oxide [9]. In previous studies, orthodontic dental bonding primers and adhesives with GO or BAG have been shown to have an anti-bacterial effect. However, data of an orthodontic adhesive with BAG@GO was lacking [4,10]. Antibacterial studies have used different methods (cloning forming unit (CFU), flow cytometry, propidium monoazide (PMA) treatment) [11,12]. The OD600 75 assay was selected due to its feasibility in dental hospital conditions. The pH cycle method was applied to evaluate tooth surface demineralization. After acid-base circulation, sample surfaces can be tested with a hardness tester, or surface opacity with cone-beam computer tomography (CBCT). In this study, CBCT has the advantage of evaluating the entire surface, so this study analyzed cone-beam CT after pH cycle [13]. Therefore, the purpose of this study was to evaluate mechanical and biological properties of orthodontic bonding adhesive enriched in graphene oxide and bioactive glass.

## 2. Materials and Methods

### 2.1. Synthesis of Bioactive Glass/Graphene Oxide

BAG was synthesized with the quick alkali-mediated sol-gel method. The procedure involved mixing and stirring of 2.8 mL of 2M NHO_3_ (Samchun, Seoul, Korea), 13.9 mL of distilled water, and 50 mL of ethanol (Samchun, Seoul, Korea) for 30 min. The mixture was then spun at 600 rpm at room temperature to produce an aqueous acid solution. A total of 21.6 mL of tetraethyl orthosilicate (TEOS, Sigma-Aldrich, St. Louise, MO, USA) was added to the aqueous acid solution and stirred for 30 min at room temperature. A total of 2.2 mL (12.95 mmol) of triethyl phosphate (TEP, Sigma-Aldrich, St. Louise, MO, USA) was added to the solution and stirred for 30 min. A total of 14.04 g of Ca(NO_3_)_2_·4H_2_O was added to the solution and stirred for 30 min to convert the mixture into a transparent sol state. 2M NH_4_OH (Samchun, Seoul, Korea) was added to the sol, and a muddler was used to prevent the sol from turning into a bulky gel state. Polycondensation reaction was induced by 24 h aging in a 60 °C oven, 24 h drying in an 80 °C oven, washing with distilled water and ethanol, and 4 h of heat processing in a 600 °C furnace. BAG@GO compound was synthesized with BAG and GO (liquid, 4 mg/mL, Graphenea Inc., Cambridge, MA, USA) by colloidal processing. BAG and GO suspensions were prepared using deionized and distilled water as a solvent, respectively, and were sonicated for 2 h in a bottle containing ice cubes. The concentration of GO (2 mL/mL) and BAG (80 mg/mL) were used to set the weight ratio of BAG and GO to 40:1. After the suspensions were sonicated, GO suspension was combined with BAG suspension through a separatory funnel under 200 rpm magnetic stirring. A mixture of the suspension was dried for 24 h in a 65 °C vacuum oven, after which another 2 h of magnetic stirring was conducted.

### 2.2. Characterization of Bioactive Glass/Graphene Oxide

The shape of the sample was analyzed with field-emission scanning electron microscopy (FESEM; SUPRA25; Oberkochen, Germany). The XRD pattern of BAG@GO was measured with an automated X-ray powder diffractometer (XRD; Ultima IV, Rigaku, The Woodland, TX, USA) with CuKα radiation of (λ = 1.5409292 Å), 40 kV, 40 mA, step size of 0.0010 deg, and a scanning rate of 1.00 deg/sec in the 2θ range of 05 to 85 deg.

### 2.3. Mechanical Properties

#### 2.3.1. Disk Preparation for Mechanical Properties

A disk (5 mm in diameter and 1 mm in height) was used to examine the physical properties of the orthodontic bonding adhesive with BAG@GO. A total of 2 mL of orthodontic bonding adhesive LV (Transbond™ Supreme Low-Viscosity Light Cure Adhesive, 3M, Monrovia, CA, USA) with 1, 3, and 5 wt.% of BAG@GO were inserted into a 2-mL Black e-tube. The adhesives with BAG@GO were shaken twice for 20 s each with a mixer (ProMix^TM^, Dentsply Caulk, York, PA, USA). The sample was mixed homogeneously, poured into a brass cast, covered with 0.2 mm thick slide glass, and light-cured with VALO (LED, Ultradent Products, South Jordan, UT, USA) for 20 s.

#### 2.3.2. Microhardness

Five disks were examined for each group of adhesives with a different weight percent of BAG@GO. A microhardness tester (MVK-H1, Akashi, Japan) was used to measure hardness Vickers (Hv), applying 200 gf of loading.

#### 2.3.3. Shear Bond Strength

Five premolars extracted for orthodontic reasons were prepared for each group. This study was reviewed and approved by the Institutional Review Board of Pusan National University Dental Hospital (PNUDH-2016-025). Any tooth with enamel defects, including caries or WSL, was excluded. The surfaces of the teeth where the brackets were intended to be applied were cleansed with prophylaxis cup and non-fluoridated pumice, washed with water for 10 s, and air-dried. 35% phosphoric acid gel (Ultra-Etch, Ultradent, South Jordan, UT, USA) was used to etch the surfaces of the teeth; after 15 s, the etchant was washed off with suction, and the teeth surfaces dried. Transbond™ XT Light cure adhesive primer (3M, Monrovia, CA, USA) was applied to the dried teeth surfaces, and gentle air blow was applied for 2 s. Premolar brackets (Arista^TM^, Farmingdale, NY, USA) were then adhered to the surface, parallel to the long axis of the tooth. Excess of the orthodontic bonding adhesive was removed, and the orthodontic bonding adhesive was light-cured for 5 s from mesial and distal directions. All procedures were performed by the recommended method for Transbond™ XT Primer. Bracket-bonded teeth were kept in distilled water for 24 h, and examined with a universal testing machine (Instron Corporation, Canton, MA, USA) afterwards; the steel rod of the machine was vertically positioned on the bracket on the tooth, and maximum load (N) was measured with crosshead speed of 1 mm/min. Measured load value, N, was divided by 11.83 mm^2^—the surface area of the bracket base, to calculate the bond strength (MPa). Bonding failure of the debonded tooth surface was evaluated with the Adhesive Remnant Index (ARI) using the following standards: 1—all the adhesive remained on the tooth; 2—more than 90% of the adhesive remained on the tooth; 3—from 10 to 90% of the adhesive remained on the tooth; 4—less than 10% of the adhesive remained on the tooth; and 5—no adhesive remained on the tooth.

### 2.4. Biological Properties

#### 2.4.1. Cell Viability Assay

The disk was disinfected in ethylene oxide (EO) gas, put in a 96-well plate, and radiated with UV light for 100 min. Human gingival fibroblasts (HGF-1) (ATCC, Rockville, MD, USA) were cultured in Dulbecco’s modified Eagle’s medium (DMEM, Hyclone Logan, UT, USA) with 10% fetal bovine serum (FBS, Hyclone Logan, UT, USA) and 100 IU/mL of penicillin/streptomycin (Hyclone Logan, UT, USA). HGF-1 was evenly divided and injected into 96-well plates containing samples, and the plates were incubated for 24 h in a 37 °C, 5% CO_2_ incubator. 5 mg/mL of MTT [3-(4,5-dimethylthiazol-2-yl)-2,5-diphenyltetrazolium bromide] (Sigma-Aldrich, USA) was injected into the cultivated samples. After the samples had undergone 4 h of reaction in a dark room, the supernatant liquid was removed from the samples, and MTT crystals were dissolved in dimethyl sulfoxide (DMSO, Sigma-Aldrich, USA, 150 µL/well). Absorbance was measured at a wavelength of 620 nm (Sunrise^TM^, Männedorf, Switzerland).

#### 2.4.2. Antibacterial Test

*Streptococcus mutans* (*S. mutans*, Seoul, Korea) used in this research was incubated with brain heart infusion (BHI) at 37 °C. The disk was made as mentioned above in the mechanical properties test. The disk was disinfected in EO gas, put in a 96-well plate, and radiated with UV light for 100 min. *S. mutans* (1.0 × 10^5^ CFU/mL, 200 μL) was inserted into the plate. Three samples of *S. mutans* were cultured in a 37 °C incubator for 24 h. The other three samples of *S. mutans* was incubated in a 37 °C incubator for 48 h. After the incubation time, 100 μL of the supernatant in the 96-well plate was transferred a clean 96-well plate. Absorbance was measured at a wavelength of 620 nm. Reading mode was normal (Sunrise^TM^, TECAN, Männedorf, Switzerland).

### 2.5. Anti-Demineralizationtest

**Figure 1:**
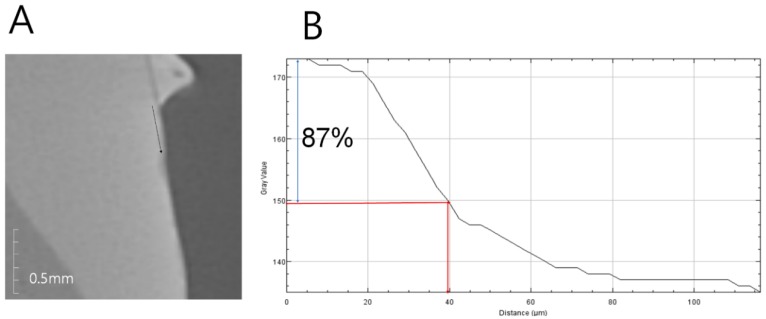
Anti-demineralization length analysis method. (**A**) Micro-computed tomography (CT) slice of ROI (region of interest) at the center of the lesion perpendicular to the enamel surface. The starting point was the end of the adhesive. Black line: the line of ROI from a reference point on the enamel surface; (**B**) Histogram in ImageJ. Blue arrow: up to 87% level of gray value from the reference point; red arrow: the distance at the 87% gray value from the reference point.

Anti-demineralization was examined using the pH cycling method, which was similar to the oral cavity acid-base dynamic cycle. Nine premolars extracted for orthodontic reasons were prepared for each group. Any tooth with enamel defects, including caries or WSLs, was excluded. Each tooth was invested in molded acrylic resin (Caulk Orthodontic Resin, Dentsply Caulk, York, PA, USA). Invested teeth samples were cleansed with prophylaxis cup and non-fluoridated pumice; they were then washed with water for 10 s and dried. Only 5 × 5 mm of tooth surface was exposed to the etchant, and all other surfaces were protected with tape. The etchant, 35% phosphoric acid gel (Ultra-etch, Ultradent, South Jordan, UT, USA), was applied for 30 s and washed off with water for 10 s, then the tooth surface was dried. Orthodontic bonding adhesive samples were produced in the same way the disks were made. After the adhesive samples were applied to the exposed teeth surfaces and light-cured for 5 s, the tape protecting the unetched surfaces was removed. The prepared tooth samples underwent a pH cycle as follows [14]. Teeth samples were stored in distilled water for 24 h and underwent a 14-day cycle of being submerged in demineralizing solution (Biosesang, Seoul, Korea) for 6 h, and in anti-demineralizing solution (Biosesang, Seoul, Korea) for 18 h; the solutions were replaced every 7 days. Each time the samples were moved from one solution to the other, they were washed with distilled water for 1 min and dried with gentle air. The samples were examined with micro-computed tomography (CT) (InspeXio SMX-90CT Plus Benchtop micro Focus X-ray, Shimadzu, Japan), 90 kV and 109 μA. The micro-CT data was analyzed with image J (National Institutes of Health, Bethesda, MD, USA), with adjustments made according to the scale bar on micro-CT [15]. The brightness histogram was selected as the test standard for identifying sound enamel; enamel tissue with up to 87% brightness was considered sound enamel, and anti-demineralization length was measured from the point where the enamel had a brightness below 87% to the end point, at which the sample orthodontic bonding primer had been applied (Figure 1).

### 2.6. Statistical Analysis

Experimental comparison between groups was performed with one-way analysis of variance test (ANOVA) and post-hoc, with Duncan’s multiple comparison test; examined properties included microhardness, shear bond strength, antibacterial test, cell viability test, and pH cycle test. ARI was verified with the Kruskal-Wallis test. All statistical analysis was performed with the R program (version 3.5.1; R Foundation for Statistical Computing, Vienna, Austria).

## 3. Results

### 3.1. Characterisation of BAG@GO

**Figure 2:**
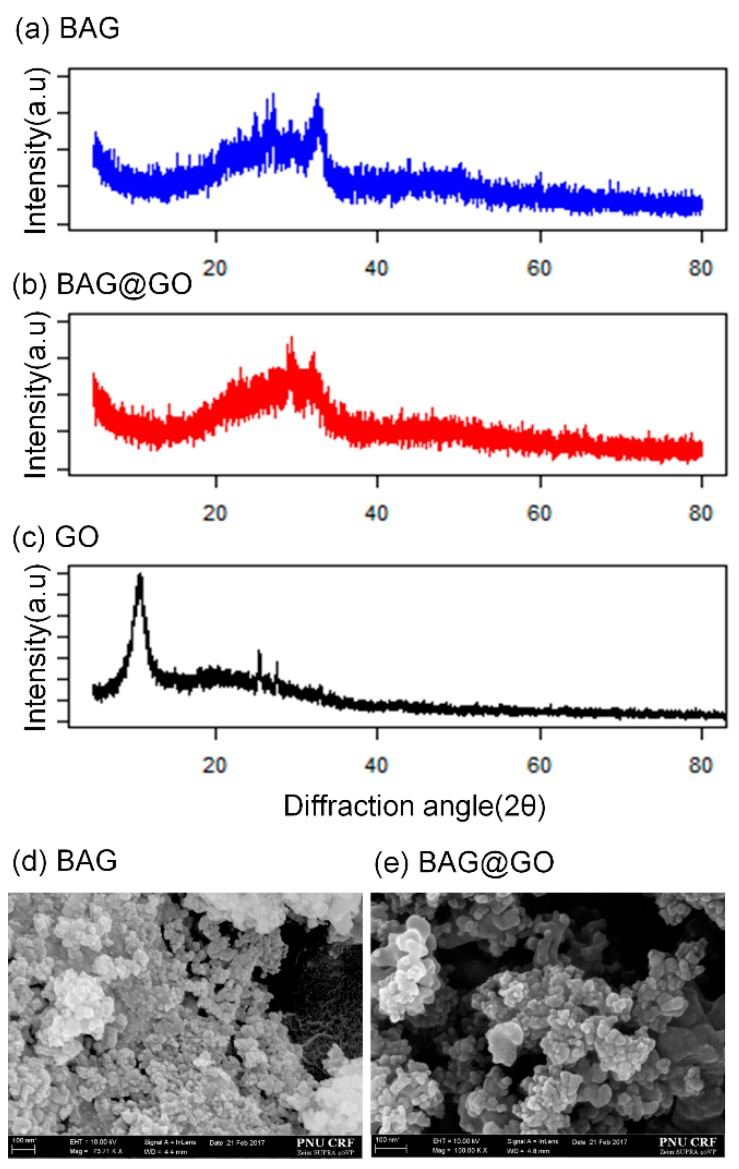
Characterization of BAG, BAG@GO and GO. (**a**) XRD of BAG; (**b**) XRD of BAG@GO; (**c**) XRD of GO; (**d**) SEM of BAG; and (**e**) SEM of BAG@GO.

BAG synthesized with sol-gel synthesis showed aggregated polygonal structure, similar to other research findings [4]. BAG@GO showed various amorphous GO layers within the BAG structure [15]. The BAG@GO SEM image showed a polygonal BAG image and also showed a plate-shaped GO. Because of the tendency of BAG to aggregate each other, BAG@GO also aggregated with each other. The XRD pattern of BAG did not show a crystalline peak. The GO XRD pattern was at 25~30° [7]. XRD patterns of BAG@GO showed combined patterns of both BAG and GO, forming an atypical pattern and a peak at 26.7° (Figure 2).

### 3.2. Mechanical Properties

#### 3.2.1. Microhardness

**Figure 3:**
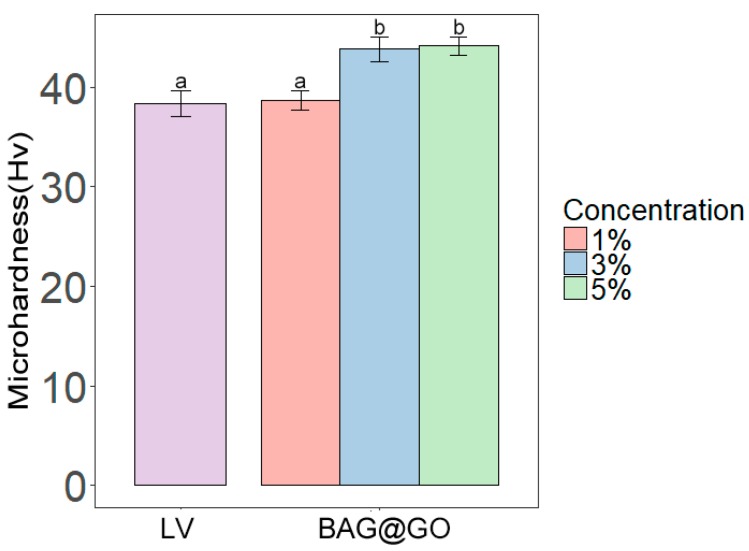
Microhardness comparison of orthodontic bonding adhesive containing LV and different weight percentages of BAG@GO. The same letters indicate no statistically significant difference between the groups (*p* < 0.05) by Duncan’s multiple comparison test. Error bars are shown ± standard deviation.

The groups with 3 wt.% (43.8 ± 5.3 Hv) and 5 wt.% (44.1 ± 4.2 Hv) of BAG@GO showed a significant increase in microhardness compared with the group of just LV. However, 1 wt.% BAG@GO group (38.6 ± 4.2 Hv) showed no increase compared with the LV group (38.4 ± 5.5 Hv) (Figure 3). The microhardness of the orthodontic bonding adhesive with BAG@GO was improved compared with the LV group.

#### 3.2.2. Shear Bond Strength

**Figure 4:**
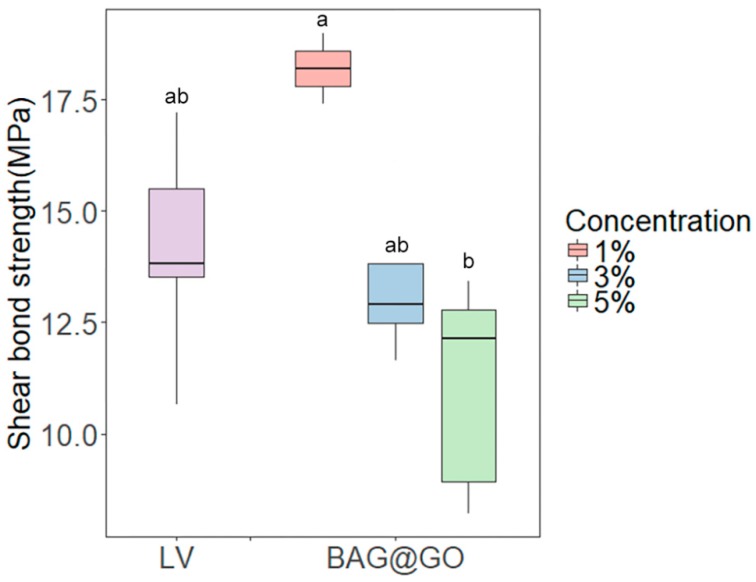
Shear bond strength comparison of orthodontic bonding adhesive containing LV and different weight percentages of BAG@GO. The same letters indicate no statistically significant difference between the groups (*p* < 0.05) by Duncan’s multiple comparison test. Error bars are shown ± standard deviation.

No statistical difference between the LV group and the sample group was found in the shear bond strength test. The shear bond strength decreased as the amount of BAG@GO in the sample increased (*p* < 0.05). The 3 wt.% (13.4 ± 1.9 MPa) and 5 wt.% (11.1 ± 2.4 MPa) BAG@GO groups showed a decreased shear bond strength compared to the LV group (14.1 ± 0.8 MPa), but this was not significantly different. The 1 wt.% BAG@GO group (18.2 ± 0.8 MPa) showed an increased shear bond strength compared to the LV group, but this was not significantly different (Figure 4).

#### 3.2.3. Adhesive Remnant Index (ARI) Score

The ARI scores of the LV group 1/5 (20%) was 3, and 4/5 (80%) was 4. The 1% wt. BAG@GO group was showed a 5/5 (100%) ARI score of 4. The ARI scores of the 3 wt.% of BAG@GO group were 3 (1/5, 20%) or 4 (4/5, 80%). The ARI scores of the 5 wt.% of BAG@GO group were 3 (3/5, 60%) or 4 (2/5, 40%). The sample group with BAG@GO did not show a different trend. No groups showed any statistically significant differences (*p* < 0.05) (Table 1). All of the LV groups and the BAG@GO groups containing orthodontic bonding adhesive showed similar bonding ability on the tooth surface.

### 3.3. Biological Properties

#### 3.3.1. Cell Viability Test

Cell viability test results after 48 h and 72 h were confirmed: control group (sterilized saline) showed significantly higher results of the LV group and the sample groups (1, 3, 5 wt.% BAG@GO). Samples with 1 wt.% BAG@GO showed higher cell viability when compared to samples with 3 and 5 wt.% BAG@GO. However, the LV group showed no statistically significant difference when compared to the 1, 3, 5% BAG@GO groups. For the test result after 48 h, the LV group (24.7 ± 4.4%) and the 1, 3, 5 wt.% BAG@GO groups (1 wt.% BAG@GO: 52.4 ± 10.1%, 3 wt.% of BAG@GO: 32.3 ± 5.0% and 5 wt.% of BAG@GO: 23.8 ± 0.8) were not significantly different. Similarly, after 72 h, there were no statistically significant differences among the LV group (11.4 ± 4.5%) and the 1, 3, 5 wt.% BAG@GO groups (1 wt.% BAG@GO: 24.7 ± 4.5%, 3 wt.% BAG@GO: 16.6 ± 3.2%, and 5 wt.% BAG@GO: 15.8 ± 2.9%).

**Figure 5:**
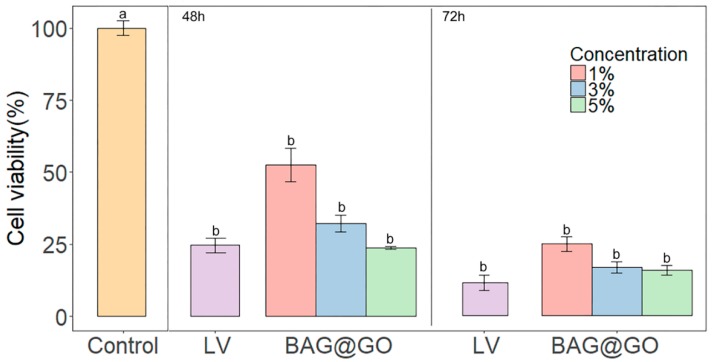
Cell viability test by HGF cytotoxicity on cured LV and BAG@GO-containing orthodontic bonding adhesive. Cell viability test results after 48 and 72 h are shown. The same letter indicates no statistically significant difference between the groups (*p* < 0.05) by Duncan’s multiple comparison test. Error bars are shown ± standard deviation.

This showed that cell viability was not higher with greater BAG@GO concentration (Figure 5).

#### 3.3.2. Antibacterial Properties

**Figure 6:**
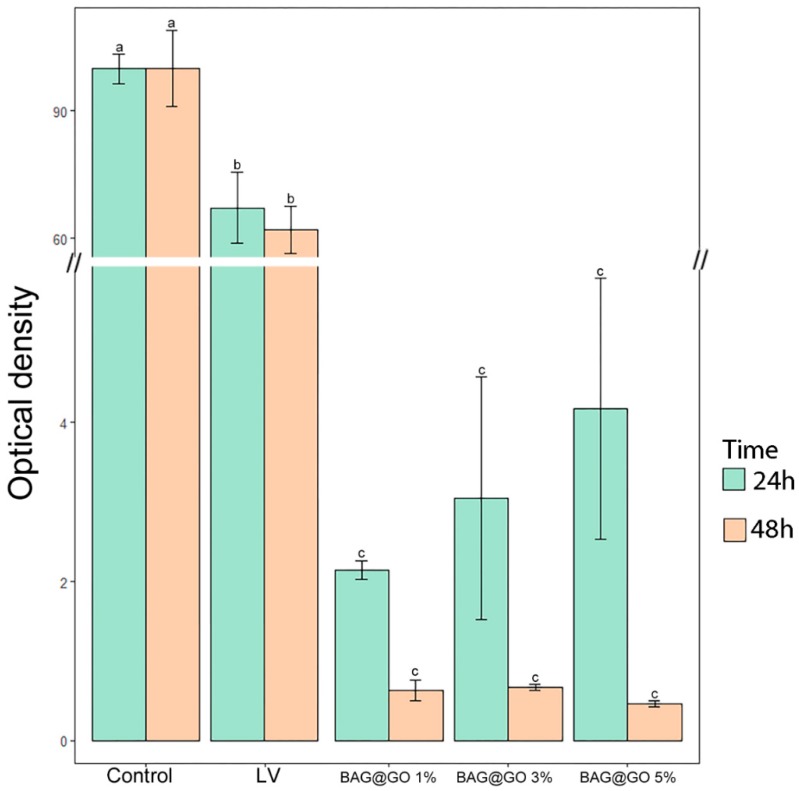
The difference in antibacterial properties between cured LV and BAG@GO orthodontic bonding pastes at 24 h. The same letter indicates no statistically significant difference between the groups (*p* < 0.05) by Duncan’s multiple comparison test. Error bars are shown ± standard deviation.

Control was 100%, and the antimicrobial effect after 24 h showed a significant difference between the LV group (67.2 ± 14.5%) and the 1, 3, 5 wt.%. BAG@GO groups (1 wt.% BAG@GO: 2.1 ± 0.2%, 3 wt.% BAG@GO: 3.0 ± 2.6%, and 5 wt.% BAG@GO: 4.2 ± 2.8%). On the 48 h test, the LV group (62.0 ± 9.8%) was not significantly different from the BAG@GO groups (1 wt.% BAG@GO: 0.6 ± 0.2%, 3 wt.% BAG@GO: 0.6 ± 0.1%, and 5 wt.% BAG@GO: 0.5 ± 0.1%). With respect to the antibacterial effect, sample groups with BAG@GO showed a significantly higher antibacterial effect compared to the control group (containing nothing) and the LV group. All groups showed higher antibacterial effect 48 h after the reaction than groups just 24 h after the reaction. The sample group with 5 wt.% BAG@GO showed the highest antibacterial effect. Groups 24 h after the reaction did not show any correlation between the concentration of BAG@GO and antibacterial effect, while groups 48 h after the reaction showed antibacterial effect proportional to the concentration of BAG@GO (Figure 6).

### 3.4. Anti-Demineralization Test

**Figure 7:**
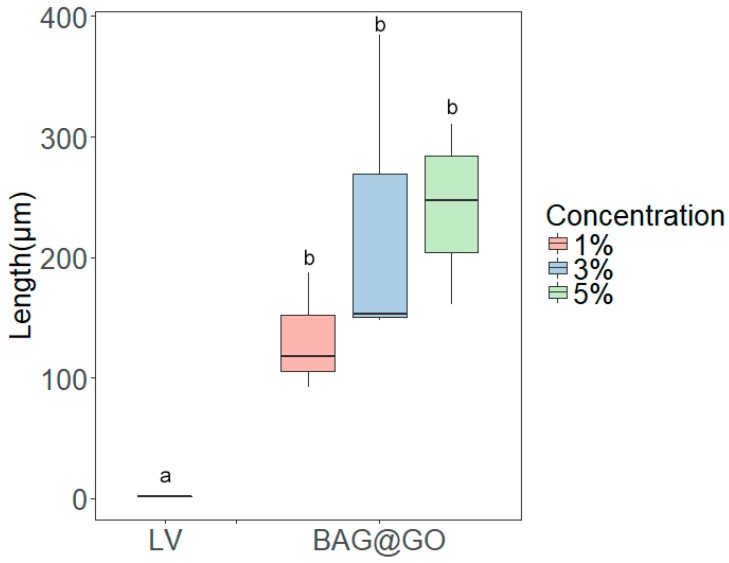
Anti-demineralization length comparison of orthodontic bonding adhesive containing LV and BAG@GO by Image J analysis. The same letter indicates no statistically significant difference between the groups (*p* < 0.05) by Duncan’s multiple comparison test. Error bars are shown ± standard deviation.

**Figure 8:**
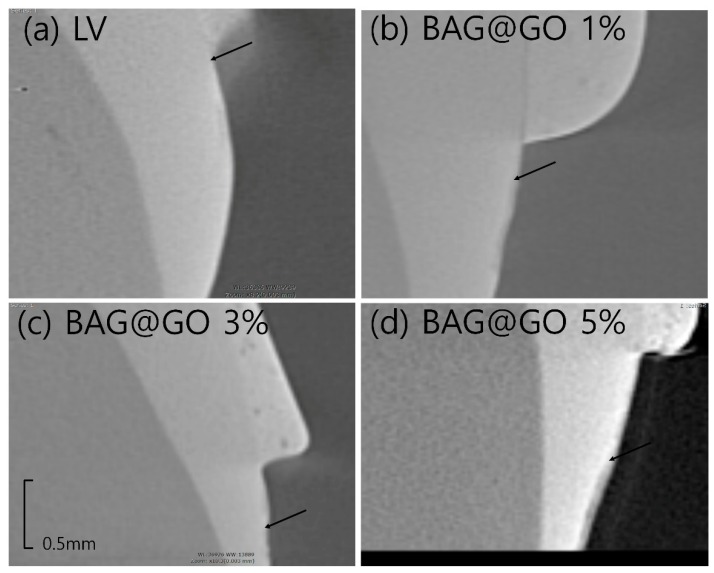
Anti-demineralization point (black arrow) of the LV and BAG@GO orthodontic bonding adhesive via CBCT. (**a**) LV; (**b**) 1 wt.% BAG@GO; (**c**) 3 wt.% BAG@GO; and (**d**) 5 wt.% BAG@GO.

The anti-demineralization length of BAG@GO-containing orthodontic bonding adhesive groups was longer than the LV group (1.3 ± 0.2 μm). Comparing among BAG@GO-containing orthodontic bonding adhesives, anti-demineralization length increased with the increase in BAG@GO concentration: 1% (132.4 ± 49.0 μm), 3% (228.7 ± 135.3 μm) and 5% (218.4 ± 57 μm). All sample groups with BAG@GO showed a statistically significantly higher anti-demineralization effect than the LV group, and the anti-demineralization effect was proportional to the concentration of BAG@GO (Figure 7 and Figure 8).

## 4. Discussion

WSLs are side effects of orthodontic treatment that significantly affect one of the most important orthodontic treatment purposes: aesthetics. Preventive treatment for WSLs that requires patient cooperation is unpredictable, and clinical fluoride application or prosthodontic treatment requires additional expense and chair time, making it difficult for orthodontists to perform effectively. Research is currently targeted at different methods of mixing biomaterials with bonding materials to prevent WSLs. These biomaterials act as fillers, and therefore do not interfere with the physical properties of the resin adhesive [4,15].

Among biomaterials, BAG was introduced to the field of dentistry due to the effect of remineralization. The mechanism of BAG in the body is the exchange of Ca^2+^ and H^+^, the O-Si-O bonds are broken, and then Si-OH (silanol) is formed on the surface. After silanol condensation reaction occurs, Ca^2+^ and PO_4_^3−^ on body fluid reacts with silanol to make amorphous calcium phosphate (ACP). Hydroxy-carbonate apatite (HCA) is created by the addition of OH^−^ and CO_3_^2−^ ions to ACP. Mineral contents of HCA are similar for bone and tooth; HCA can combine with bone or tooth [16]. GO is a compound of carbon and oxygen; the structure of GO is a single-layer plate. GO has been studied as a new biomaterial in dentistry due its antimicrobial effect against various pathogens. GO has low toxicity, so it is suitable for use as a biomaterial in dentistry [9,17].

In this study, an orthodontic adhesive with 1 wt.% of BAG@GO showed no statistically significant difference in micro-hardness when compared to existing orthodontic adhesives, but those with 3 wt.% or 5 wt.% of BAG@GO showed a significant increase in micro-hardness. This result corresponds with the results of the research performed by Lv et al.; composites with graphene nano-sheet and 0.5 wt.% or 1.0 wt.% HA showed a 30–40% increase in microhardness, and PMMA with 0.5, 1.5, 2.0 wt.% nano-GO showed an increase in Vicker’s hardness [10,18]. This is because the stable layer structure of GO acts as an effective filler in the material, subsequently increasing the microhardness.

The shear bond strength of the LV group showed no statistically significant difference compared with the BAG@GO group, while adhesives with 1 wt.% BAG@GO showed a slight increase in shear bond strength; the increased SBS is due to physical interlocking, because BAG or BAG@GO adhesives create a tough, irregular surface (300–400 nm) [18]. The surface irregularity also hinders crack propagation in the material by functioning as an interlocking bridge between cracks. The adhesive group with 5 wt.% BAG@GO showed lower SBS, but this result was not statistically significant. This may be attributable to a decrease in brightness resulting in a lower curing rate; in clinical use, a longer curing time is necessary when this material is applied.

All sample groups containing BAG@GO showed a statistically significant increase in antibacterial activity compared with the control group (sterilized water) and the LV group. The 48 h group showed higher antibacterial effect than the 24 h group, implying the higher effectiveness of BAG@GO samples over time. Bioactive glass exerts antibacterial activity by decreasing PO4, so previous study showed the possibility to inhibit bacterial metabolism, subsequently arresting enamel demineralization and decreasing the incidence of WSLs [19]. GO demonstrates its antibacterial activity via two main mechanisms: by generating reactive oxidative stress [17], where the GO nanosheet causes damage to the cell membrane, and therefore interferes with bacterial metabolic activity [20]; and by trapping and isolating the bacterial cell surface from the environment, thus blocking the membrane activity site [21]. These mechanisms both decrease cell metabolic activity; therefore, with time, its antibacterial effect increases. This supports the result that the 48 h group of BAG@GO showed higher antibacterial activity than the 24 h group. Research shows that GO is effective against dental pathogens, as well as general bacteria [9,10].

The chemical anti-demineralization effect through pH cycle was higher in the sample groups with BAG@GO than in the control group; a higher anti-demineralization effect was observed as the concentration of BAG@GO increased. This is because the chemical anti-demineralization effect is a result of the buffering effect of ions released by BAG, which prevents a decrease in intraoral pH [22]. The group with 1 wt.% of BAG@GO showed high efficacy, whereas groups with 3 wt.% and 5 wt.% of BAG@GO did not show any statistically significant difference.

The grain size of BAG remains limited because graphene hinders the growth of BAG grain, allowing the grain to have a more exposed contact surface with bodily fluids and to release more ions [23,24]. Sample groups with more than 3 wt.% of BAG, however, are less affected by the size of the BAG grains, because the difference in the amount of released ions increases.

In conclusion, combining BAG@GO with orthodontic adhesives will decrease WSLs because of the ion-releasing effect of BAG and the antibacterial activity of GO, and will be clinically acceptable considering the results obtained for the cell viability and mechanical properties of the samples. Orthodontic treatment, however, requires more than a year of device application. Therefore, long-term experimental results and in vivo testing in conditions similar to those found in the oral environment will be required.

## 5. Conclusions

1. Mechanical properties of orthodontic bonding adhesive enriched in graphene oxide and bioactive glass were appropriate for clinical application.
2. Biological properties of orthodontic bonding adhesive enriched in graphene oxide and bioactive glass were safe for application to patients.
3. More studies, especially in vivo studies, are needed to extensively test orthodontic bonding adhesive containing BAG@GO.

## Figures and Tables

**Table 1:** Adhesive Remnant Index (ARI) scores of tested orthodontic bonding adhesives.

Sample	LV	BAG@GO			Significant
		1%	3%	5%	
Mean (SD)	3.8 (0.4)	4.0 (0.0)	3.8 (0.4)	3.4 (0.5)	Not significant
Median, Q1–Q3	4, 4–4	4, 4–4	4, 4–4	3, 3–4	
Min.–max.	3–4	4–4	3–4	3–4	

The ARI score is not significantly different according to the Kruskal-Wallis test at α = 0.05 (n = 5). Score 1—all the adhesive remained on the tooth; score 2—more than 90% of the adhesive remained on the tooth; score 3—from 10 to 90% of the adhesive remained on the tooth; score 4—less than 10% of the adhesive remained on the tooth; score 5—no adhesive remained on the tooth.
